# Initial experience of transoesophageal echocardiography-guided percutaneous pulsed field ablation of atrial fibrillation

**DOI:** 10.1136/openhrt-2025-003172

**Published:** 2025-04-05

**Authors:** Jun Liu, Min Tang, Guodong Niu, Chao Li, Daoliang Zhang, Yong Jiang, Yan Yao, Xiang-Bin Pan

**Affiliations:** 1 Fuwai Hospital State Key Laboratory of Cardiovascular Disease, Beijing, China; 2 Fuwai Shenzhen Hospital,Chinese Academy of Medical Sciences, Shenzhen, People's Republic of China; 3 Fuwai Yunnan Cardiovascular Hospital, Kunming, Yunnan, China

**Keywords:** Atrial Fibrillation, Echocardiography, Electrocardiography, Catheter Ablation

## Abstract

**Objective:**

Pulsed-field ablation (PFA) is a new technology of catheter ablation for atrial fibrillation (AF). This research is to investigate the feasibility of a new strategy (transoesophageal echocardiography-guided pulsed field ablation, TEEP) to guide PFA for AF with no contrast and zero fluoroscopy.

**Methods:**

Patients with AF underwent TEEP under general anaesthesia with the guidance of three-dimensional (3D) transoesophageal echocardiography (TEE) throughout the procedure. After a successful transseptal puncture, the PFA catheter (CardiPulse) was delivered to the different pulmonary veins sequentially for standard PFA, and the pulmonary vein electrical isolation (PVI) was observed in real-time. After the ablation, left atrial bipolar voltage mapping under sinus rhythm was performed to verify the PVI.

**Results:**

10 patients with AF were enrolled, including 6 patients with paroxysmal AF and 4 patients with persistent AF. The mean operative time was 99±14 min, the mean time of the left atrial manoeuvre was 66±23 min, and the mean PFA ablation time was 105±8 s. First-pass PVI of all veins was achieved in all patients, thus no additional PFA applications were needed after the initial set. No contrast was needed and no X-ray was exposed. No complications were observed.

**Conclusions:**

We report the preliminary application of 3D TEE-guided PFA for AF in the world. Its immediate safety and efficacy are promising. Compared with traditional PFA procedures, TEEP has many advantages, including accuracy of the transeptal puncture, direct visualisation of contact between the catheter and myocardial tissue, no contrast and zero fluoroscopy.

**WHAT IS ALREADY KNOWN ON THIS TOPIC**

- Current pulsed-field ablation (PFA) ablation procedures are not possible without intraoperative X-ray exposure and contrast agents.

**WHAT THIS STUDY ADDS**

- PFA procedures can be performed under the guidance of transoesophageal echocardiography.

**HOW THIS STUDY MIGHT AFFECT RESEARCH, PRACTICE OR POLICY**

- The PFA procedure can be completely zero-radiation and zero-contrast-agent.

## Introduction

 Pulsed electric field ablation (PFA) has emerged as a new approach to catheter ablation of atrial fibrillation (AF). PLEASE study has confirmed the safety and efficacy of the PFA catheter (CardiPulse).^1^ The traditional PFA procedure is commonly performed under X-ray fluoroscopy, and it requires angiography with contrast agents to determine the position of the pulmonary venous (PV) antrum as well as the good contact between the PFA catheter and the PV antrum. Based on our team’s profound experience in echocardiography-guided interventional procedures for different cardiovascular diseases,^2^,^5^ we propose a new concept, namely, Transoesophageal Echocardiography-guided Pulsed field ablation (TEEP), to complete PFA procedure for AF with no X-ray exposure, no contrast and complete thorough transoesophageal echocardiography (TEE) guidance. In this small case series, we explored the safety, efficacy and feasibility of TEEP for AF.

## Methods

### Study population

10 patients with AF between July and December 2024 were treated with PFA. Preoperative enhanced CT and TEE were performed to exclude thrombosis. Routine biochemistry, ECG, ambulatory ECG and transthoracic echocardiography (TTE) were performed preoperatively. Uninterrupted oral novel anticoagulant treatment in the perioperative period was used in our previous report.^6^

### TEEP procedure

All patients signed an informed consent for the procedure before the procedure. All patients received a full therapeutic heparin dose once femoral vein vascular access with a target-activated clotting time >300 s. After successful general anaesthesia, a transoesophageal cardiac three-dimensional (3D) probe was placed to guide the whole procedure, including catheter implantation, transeptal puncture, and delivery of PFA catheter into the left atrium (LA) and different PV. A 10-polar coronary sinus electrode and a right ventricular temporary pacing electrode were implanted via the left femoral vein. The long sheath was used when left iliac vein tortuosity was encountered. To determine whether the electrodes were inside the coronary sinus or ventricle according to the ultrasound images and the ECG signals of the atria and ventricles recorded by the electrodes.

The TEE-guided transseptal puncture was performed as previously described using an SL1 long sheath and a transseptal needle^5^ (see detail in online supplemental video 1). The TEE probe was delivered to the level of the middle oesophagus (ME) and adjusted the orientation of the transducer angle by 90° to allow clear visualisation of the LA, the right atrium (RA), the superior vena cava and the inferior vena cava. Keeping the tip end of the J-guidewire, the transseptal puncture sheath and guidewire were simultaneously pulled down until they dropped to the fossa ovalis (FO) level, thus exchanging the transseptal puncture needle. On the biplane of the TEE image, the transeptal puncture sheath was rotated clockwise, forming the ‘tenting’ sign at the tip, to ensure that the needle was directed at the posterior wall of the LA and avoided pointing at the ascending aorta. The needle and sheath were pushed successively into the LA, and the 3D TEE imaging confirms the successful completion of the transseptal puncture. After the withdrawal of the inner sheath, a double-track sign of sheath formation was seen on the TEE image. Thereafter, access from the RA to the LA was obtained by the transseptal wire.

By adjusting the TEE transducer with an angle of 30°, the left superior pulmonary vein (LSPV) and the left atrial appendage are displayed. The deflectable sheath (Hangzhou Dinova EP Technology) was exchanged and the PFA catheter (CardiPulse, Hangzhou Dinova EP Technology) was delivered into the ostium of the LSPV after TEE confirmation of the guidewire’s entry into LSPV. The PFA catheter was released with a ‘basket’ configuration, and the ablation began to start after TEE confirmed that the PFA catheter was in complete contact with the ostium of LSPV. Then the catheter was retracted, the PFA catheter was adjusted to a ‘flower’ configuration, and the PFA ablation was repeated after TEE confirmed that the catheter was completely adherent to the antrum of LSPV^1^ (see detail in online supplemental video 2). The PFA energy was delivered with different pulse intensities at the PV ostium (1800 V) and PV antrum (2000 V) for ablation, three deliveries were applied at each site, and then the catheter was rotated to a new site to ensure that any gap between electrodes was fully covered. Generally, two to three ostial sites and three antral sites were required to achieve full circumferential isolation of the PVs.

Keep the angle of the TEE transducer unchanged, and push the probe to reveal the left inferior pulmonary vein (LIPV) orifice and mitral annulus. Adjust the curve of the deflectable sheath until the tip of the sheath is pointing towards the orifice of the LIPV. The guide wire was initially inserted into the LIPV, followed by the PFA catheter along the guide wire into the LIPV. Using the same strategy, the PFA was delivered sequentially to the antrum and the ostium of LIPV (see online supplemental video 3).

By adjusting the TEE transducer with an angle of 65° at the level of the ME, the right superior pulmonary vein (RSPV) and the right inferior pulmonary vein (RIPV) can be displayed. Keeping the deflectable sheath lightly bent, rotate the sheath clockwise about 90°, then the tip of the sheath can be pointed towards the orifice of the RSPV. Using the same strategy, the PFA was delivered sequentially to the antrum and the ostium of RSPV (see online supplemental video 4).

Keep the TEE probe angle and position unchanged. Bend the deflectable sheath approximately 90°, rotate the sheath counterclockwise approximately 30°, and slightly retract the sheath so that the sheath is pointing towards the RIPV. 3D TEE images can clearly show the anatomical relationship between the RSPV and RIPV, in addition to the degree of adherence of the PFA catheter to the orifices of the PVs, as well as the movement of the PFA catheter from the RSPV to the RIPV (see online supplemental video 5).

**Figure 1:**
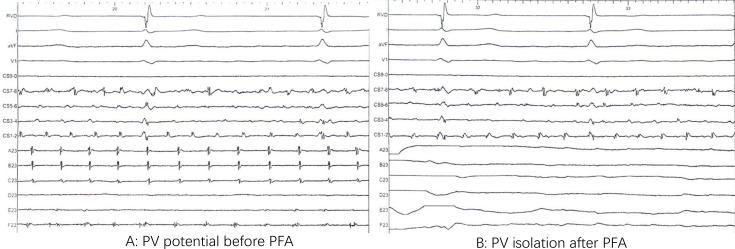
Preablation and postablation PV potential. After one pulsed field application (PFA), the disappearance of PV potentials (**B**) compared with preablation (**A**). PV, pulmonary venous.

**Figure 2:**
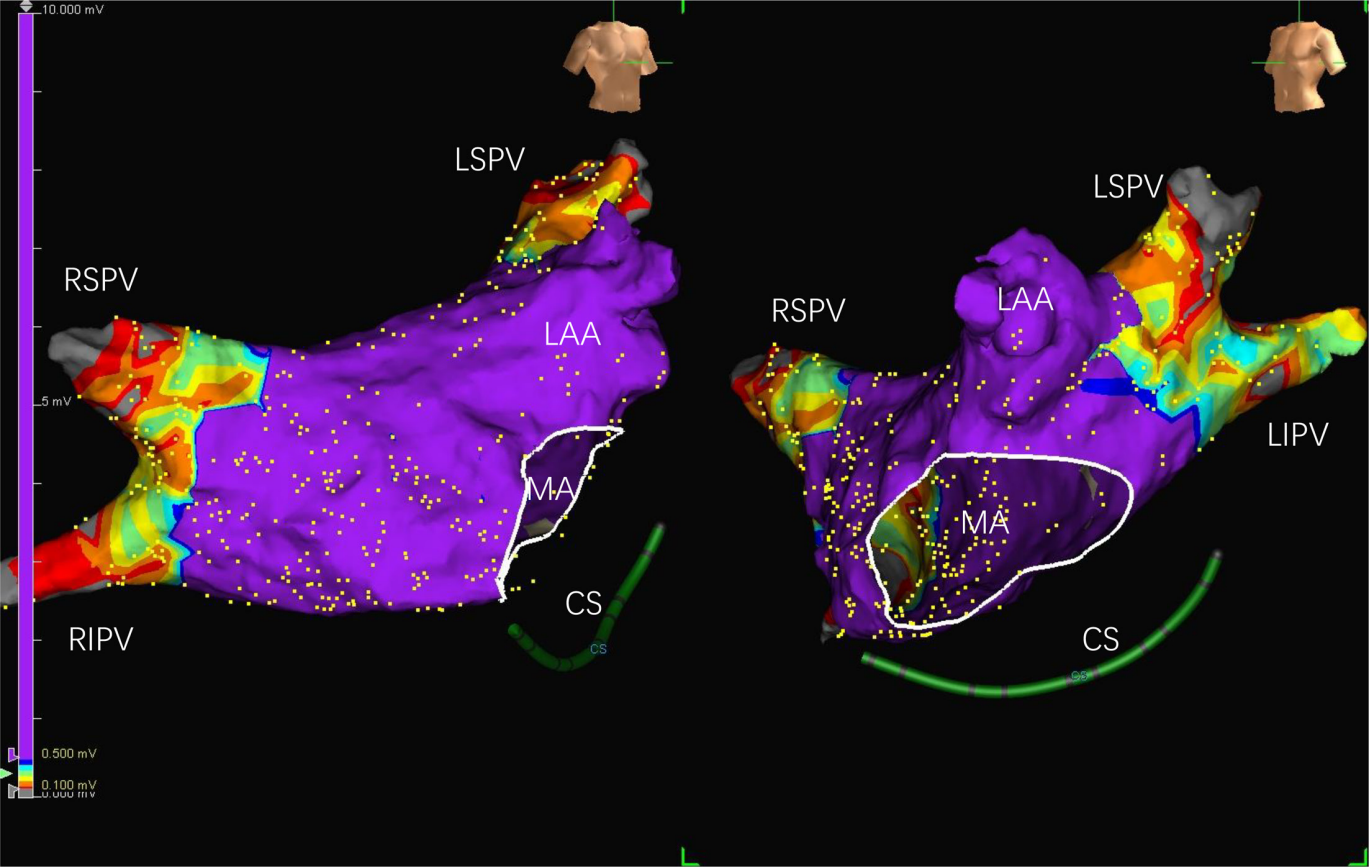
Postablation LA voltage maps. (A) Anteroposterior 3D electroanatomical views of the LA preablation (left panel) and postablation (right panel). (B) A right anterior oblique position. The colour purple is bipolar voltage >0.5 mV, and the colour grey is bipolar voltage <0.1 mV. LA, left atrium; LAA, left atrial appendage; LIPV, left inferior pulmonary vein; LSPV, left superior pulmonary vein; MA, mitral annulus; RIPV, right inferior pulmonary vein; RSPV, right superior pulmonary vein.

The pulmonary vein electrical isolation (PVI) was monitored in real time during the procedure. After the ablation was completed, electrical conversion was performed if the heart rhythm was still AF. Acute PVI was defined as no PV potentials within the targeted PV and entrance/exit conduction block at each PV after a 20 min waiting period. Pulmonary vein entrance block is confirmed in all patients with disappearance or dissociation of PV potentials recorded using the PFA catheter (see detail in figure 1). Postelectroanatomical bipolar voltage mapping was performed for further confirmation of acute PVI using the EnSite (Abbott) 3D mapping system (see detail in figure 2). If any residual PV connection was detected, additional lesions were placed. Then, the sheath was removed and the patient was awakened and returned safely to the ward.

Key ablation parameters, including total procedure time and total ablation time of PFA applications, were meticulously recorded. Adverse reactions attributable to PFA, such as cough, pain and skeletal muscle contraction, were also monitored, along with other perioperative complications.

### Postoperative management

A repeat TTE for pericardial effusion was performed after 4–6 hours. The patients were discharged from the hospital after 24 hours postoperatively if there were no interventional-related complications and were followed up for observation on outpatient basis.

### Statistical analysis

Continuous variables that conformed to normal distribution were expressed as mean±SD, and comparisons between variables were made using the Student’s t-test. Discrete variables were expressed as ratios, and the χ^2^ test or Fisher’s exact probability test was applied. Statistical analyses were performed using SAS statistical software (V.9.1).

## Results

### Patient characteristics

10 patients were enrolled. The mean age was 59±10 years, 7 (70%) males, the mean BMI was 26.9±3.0, 6 (60%) patients with paroxysmal AF, 6 (60%) patients with hypertension, 2 (20%) patients with hyperlipidaemia, 1 (10%) patient with coronary artery disease and 1 (10%) patient with sleep apnoea syndrome. Preoperative TTE showed an average LA diameter of 40.0±3.7 mm, an average left ventricular end-diastolic diameter of 47.0±5.1 mm, an average LVEF of 61.1%±6.7%, an average eGFR value of 75.1±14.0 and an average NTproBNP of 432±52 pg/mL (see detail in online supplement data).

### Outcome

The mean operative time was 99±14 min, the mean time of the left atrial manoeuvre was 66±23 min, and the mean PFA ablation time was 105±8 s. First-pass PVI of all PVs was achieved in all patients, thus no additional PFA applications were needed after the initial set. Procedure times in our initial case series were comparable to our previously reported data on PFA (99±14 min vs 123.5±38.8 min).^1^ No contrast was needed and no X-ray fluoroscopy was exposed. No complications were observed.

## Discussion

In the present study, we found that it is feasible to TEEP for AF patients. Its immediate safety and efficacy are promising. In addition, compared with traditional PFA procedures, TEEP has many advantages, including accuracy of the transeptal puncture, direct visualisation of contact between the catheter and myocardial tissue, no contrast and zero fluoroscopy.

PFA ablation for AF has the unique advantages of myocardial tissue-specific damage, fast speed of ablation and short procedure time. The choice of septal puncture site is a crucial first step for successful AF ablation procedures.^7^ The TEE-guided transeptal puncture can not only avoid the occurrence of transeptal puncture complications but also provide a precise selection of the puncture site. Based on our previous experience with transeptal puncture sites in cryoballoon ablation,^8^ we chose an anterior–inferior puncture site, mainly in consideration of the operational requirements for the RIPV. The RIPV is the current difficulty of PFA ablation operation. If the puncture site is posterior, the space between the puncture point and the RIPV is limited, and it is difficult for the catheter to be in place or the poor coaxiality between the catheter and the RIPV, which in turn causes difficulties in PVI or increases the probability of generating a gap in electrical reconduction.^9^ We chose the TEE to guide the transeptal puncture point in all of this study, which met the clinical requirement of achieving a more anterior–inferior transeptal puncture site (see online supplemental video 1), and the PFA catheters all reached the RIPV smoothly with the guidance of 3D TEE image (see online supplemental video 5).

**Figure 3:**
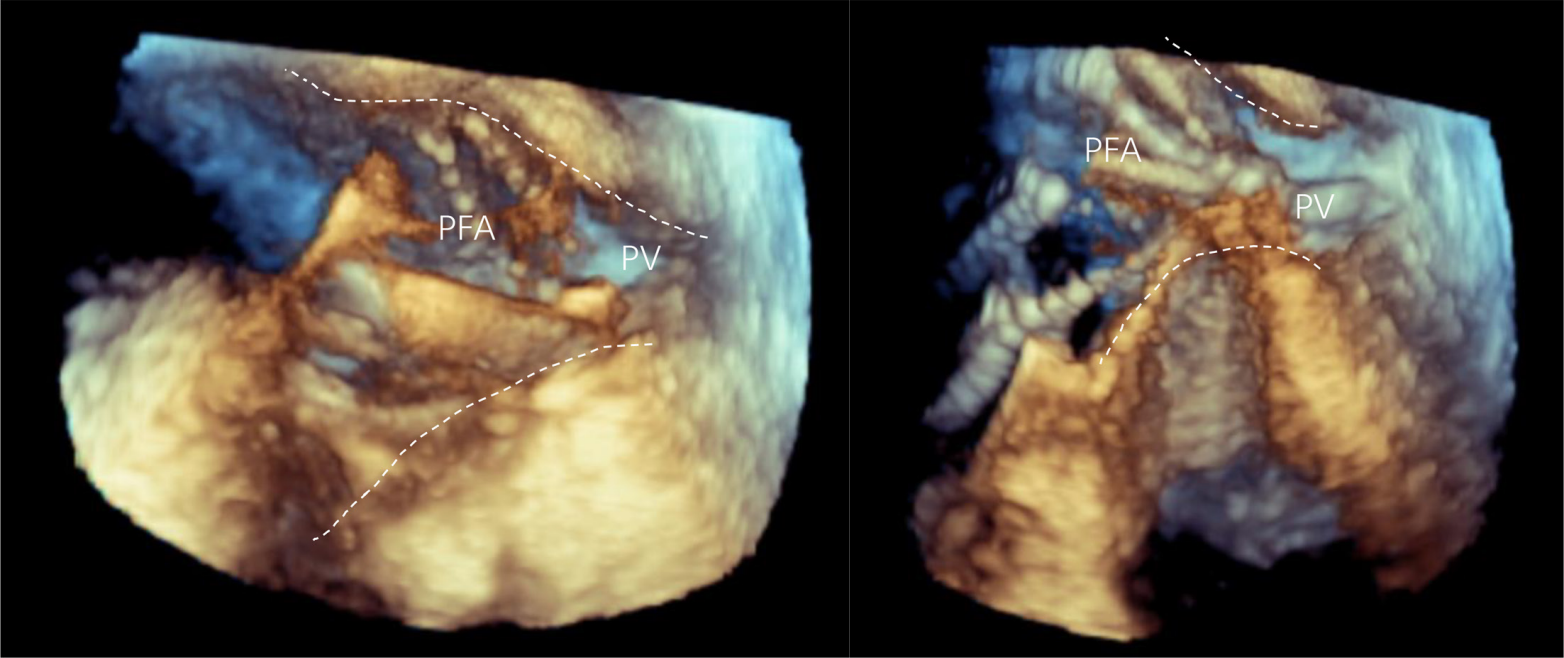
The contact between the PV ostium and PFA catheter with different morphology. The left shows the PFA catheter with basket morphology, right shows the PFA catheter with flow morphology. See the movie in online supplemental video 6. PFA, pulsed-field ablation; PV, pulmonary venous.

**Figure 4:**
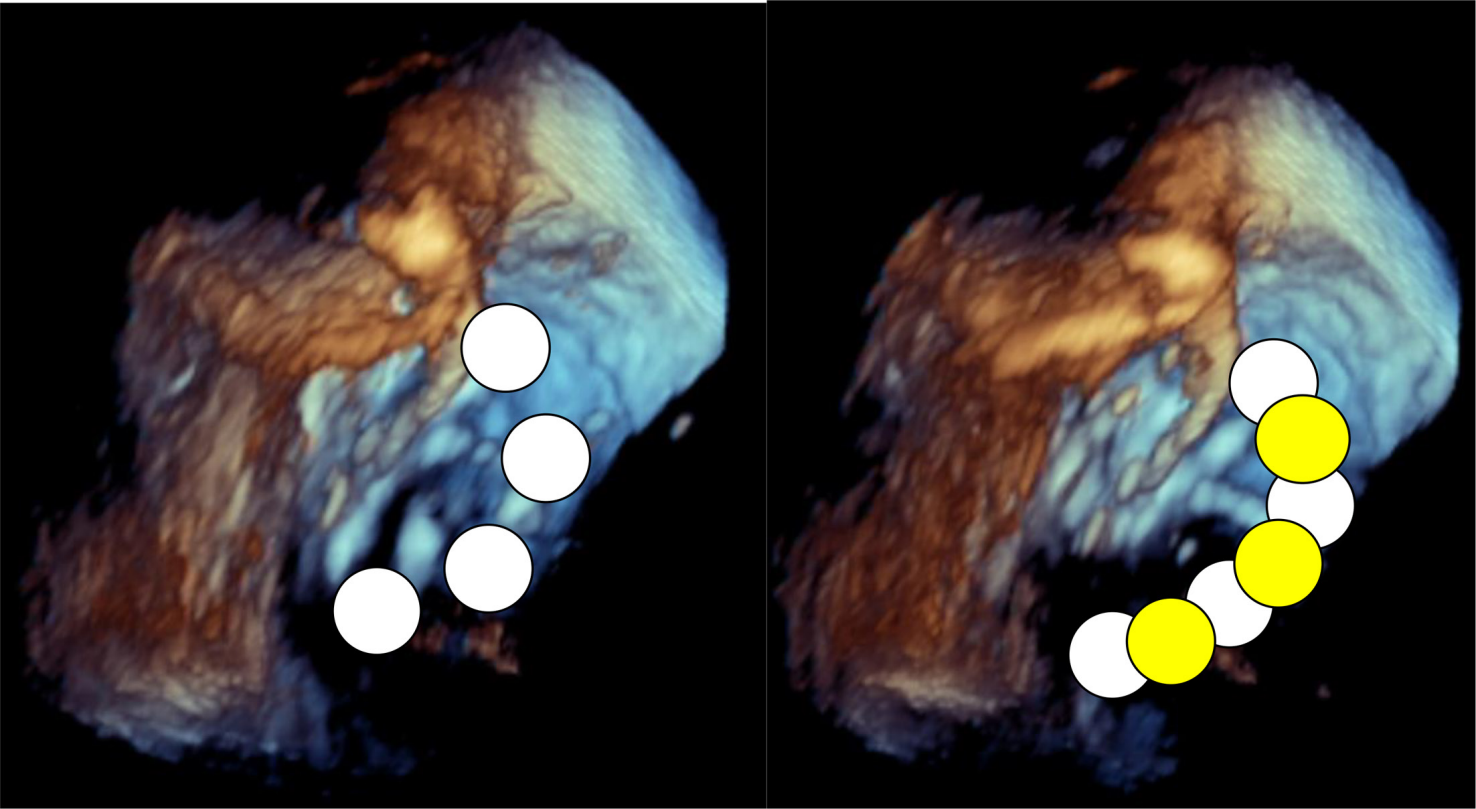
3D TEE-guided PFA catheter rotation to overlap the targeted ablation area. The left side shows the lesion of the previous PFA with white dots, and the right side shows the lesion of the next PFA with yellow dots. See the movie in the online supplemental video 7. PFA, pulsed-field ablation; TEE, transoesophageal echocardiography.

The good contact between the catheter and the myocardial tissue is critical for the biological effects of catheter ablation, and the PFA is no exception.^10^ Radiofrequency catheter ablation can determine the degree of adherence using a pressure indicator^11^ or photoreceptor^12^ at the catheter tip. Cryoballoon ablation determines the degree of balloon-to-tissue adherence by contrast leakage after selective PV angiography.^13^ Currently, PFA catheters cannot be designed using radiofrequency ablation or cryoablation and rely mainly on the spatial position of the catheter under the X-ray exposure^14^ or difference of impedance measurement^1^ to determine contact between the PFA catheter and tissue. However, the above methods are not direct judgements and have certain defects. The impedance value is measured as the circuit impedance which does not reflect the local impedance. And, local impedance is influenced not only by the contact force but also by the catheter orientation angle.^15^ The use of TEE, on the other hand, enables direct visualisation of the extent of catheter-tissue contact, especially with real-time 3D TEE technology. The major advantages of 3DTEE technology include that when on the longitudinal projection plane of the PV antrum, the contact between the PV tissue and the PFA catheter can be visualised in different morphological states, either with basket type or with flower type (see example in figure 3 and video 6 in online supplemental file). When on the transverse projection plane of the PV antrum, the target lesion can be visualised when they cross each other after rotating the PFA catheter (see example in figure 4 and video 7 in online supplemental file). Overlapping is the most common manipulation of PFA catheters to reduce the occurrence of PV gaps. Neither PFA catheter position marking with the help of a 3D mapping system nor 2D image from ICE technology is comparable to 3DTEE with distinguishing direct.

The ALARA (as low as reasonably achievable) principle is based on the hypothesis that there is no threshold below which ionising radiation is free from harmful effects for the operator and the patient. Traditional PFA procedures require pulmonary venography to clarify the location of the pulmonary veins and catheter location. Intraoperative X-ray exposure and the use of contrast media are also required, which can be detrimental to the health of both the physician and the patient. The use of TEE-guided PFA can avoid the damage from the X-rays for both doctors and patients and the contrast for the patient. 3D mapping systems help to decrease the dependence on radiography and contrast but require expensive equipment and technician support. We used the TEE guidance to complete the PFA procedure, achieving: (1) no X-ray exposure at all, avoiding radiation damage to both the physician and the patient. Several studies have demonstrated the adverse effects of radiation, including skin injury, cataracts, decreased fertility and malignancies.^16^ (2) No contrast agent at all, especially suitable for patients with a known history of contrast allergy or with potentially allergic contrast agents. An estimated 300 million persons worldwide have asthma. Asia is the world’s most populous continent, with a population of almost 4 billion people and many emerging economies.^17^ (3) No contrast agent also can decrease the risk of postprocedural contrast agent-related kidney disease, especially in patients with combined renal insufficiency. This is even more important for PFA surgery. It has been reported that the phenomenon of haemolysis caused by PFA can lead to acute renal failure.^18^ Some studies have reported the value of intracardiac echocardiography in PFA ablation, but the position of intracardiac ultrasound catheters in the cardiac chambers is not easy to fix, and it is susceptible to the influence of other catheters.^19^ The 3D-TEE guidance that we used in this study can overcome this shortcoming and give more room for manipulation of the PFA catheter. (4) Considering that the availability of TEE far exceeds that of the expensive ICE and 3D mapping systems, TEE-guided PFA techniques are more promising and more beneficial to the majority of patients, especially in economically poorer regions and developing countries.

## Limitations

The number of cases in this study was small, the follow-up period was short, and our ongoing clinical trials will confirm the long-term efficacy and safety of this approach. In addition, we will also compare the differences between TEE-guided and ICE-guided procedures to further evaluate the clinical value of this procedure.

## supplementary material

10.1136/openhrt-2025-003172 online supplemental file 1

10.1136/openhrt-2025-003172 online supplemental file 2

10.1136/openhrt-2025-003172 online supplemental file 3

10.1136/openhrt-2025-003172 online supplemental file 4

10.1136/openhrt-2025-003172 online supplemental file 5

10.1136/openhrt-2025-003172 online supplemental file 6

10.1136/openhrt-2025-003172 online supplemental file 7

10.1136/openhrt-2025-003172 online supplemental file 8

## Data Availability

Data are available in a public, open access repository.
