# Supplementary material for: Initial experience of transoesophageal echocardiography-guided percutaneous pulsed field ablation of atrial fibrillation
Source: Open Heart. 2025 Apr 5;12(1):e003172. doi: 10.1136/openhrt-2025-003172 (PMC11973749; doi:10.1136/openhrt-2025-003172)
Supplement: online supplemental file 1 [file openhrt-12-1-s001.docx]

Supplement data

| Table 1 includes patients’ characteristics. | | | | | |  |  |  |  |  |  |
| --- | --- | --- | --- | --- | --- | --- | --- | --- | --- | --- | --- |
|  | P1 | P2 | P3 | P4 | P5 | P6 | P7 | P8 | P9 | P10 | Average |
| Age(years) | 51 | 60 | 52 | 47 | 60 | 49 | 67 | 79 | 66 | 56 | 59±10 |
| Sex-male | M | F | M | M | F | M | F | M | M | M | 7/70.0% |
| Body mass index(kg/m^2^) | 21.6 | 28.0 | 25.8 | 30.9 | 27.4 | 25.3 | 32.3 | 24.2 | 26.4 | 271. | 26.9±3.0 |
| AF type | PerAF | ParAF | ParAF | PerAF | ParAF | PerAF | PerAF | ParAF | PerAF | ParAF | 6/60.0% |
| Hypertension | + | + |  | + |  | + | + |  |  | + | 6/60.0% |
| Coronary artery disease |  | + |  |  |  |  |  |  |  |  | 1/10.0% |
| HC | + |  |  | + |  |  |  |  |  |  | 2/20.0% |
| OSAS |  |  |  | + |  |  |  |  |  |  | 1/10.0% |
| LA diameter(mm) | 42 | 45 | 38 | 38 | 40 | 36 | 47 | 40 | 39 | 35 | 40.0±3.7 |
| LVED(mm) | 41 | 57 | 43 | 43 | 48 | 46 | 53 | 48 | 42 | 49 | 47.0±5.1 |
| LVEF, % | 55 | 65 | 60 | 62 | 61 | 55 | 55 | 57 | 77 | 64 | 61.1+6.7 |
| eGFR(ml/min) | 76.6 | 87.3 | 66.2 | 86.2 | 62.7 | 73.8 | 50.8 | 67.3 | 88.4 | 90.5 | 75.1±14.0 |
| NTproBNP(pg/ml) | 1576 | 1206 | 212 | 200 | 87 | 290 | 230 | 240 | 250 | 31 | 432±52 |
| Total procedure time(min) | 110 | 85 | 90 | 100 | 100 | 90 | 80 | 90 | 120 | 120 | 99±14 |
| PFA catheter dwell time(min) | 70 | 43 | 50 | 60 | 89 | 70 | 31 | 60 | 100 | 100 | 66±23 |
| Total PFA time(sec) | 115 | 105 | 100 | 105 | 100 | 95 | 100 | 105 | 100 | 120 | 105±8 |
| Acute PVI success | 100% | 100% | 100% | 100% | 100% | 100% | 100% | 100% | 100% | 100% | 100% |
| Fluoroscopy time (s) | 0 | 0 | 0 | 0 | 0 | 0 | 0 | 0 | 0 | 0 | 0 |
| Contrast (ml) | 0 | 0 | 0 | 0 | 0 | 0 | 0 | 0 | 0 | 0 | 0 |
| Abbreviations: BMI, body mass index; LVEF, left ventricular ejection fraction; NT-proBNP, N-terminal pro-B-type natriuretic peptide; GFR, glomerular filtration rate. ParAF Paroxysmal atrial fibrillation PerAF persistent atrial fibrillation HC，hypercholesterolaemia；OSAS，obstructive sleep apnea syndrome. | | | | | | | | | | | |
